# Supplementary material for: The causal relationship between the human gut microbiota and pyogenic arthritis: a Mendelian randomization study
Source: Front Cell Infect Microbiol. 2024 Nov 26;14:1452480. doi: 10.3389/fcimb.2024.1452480 (PMC11629706; doi:10.3389/fcimb.2024.1452480)
Supplement: Supplementary file 1 [file DataSheet1.zip › mendelian test/ebi-a-GCST90027480.csv_leave_one_out.pdf]

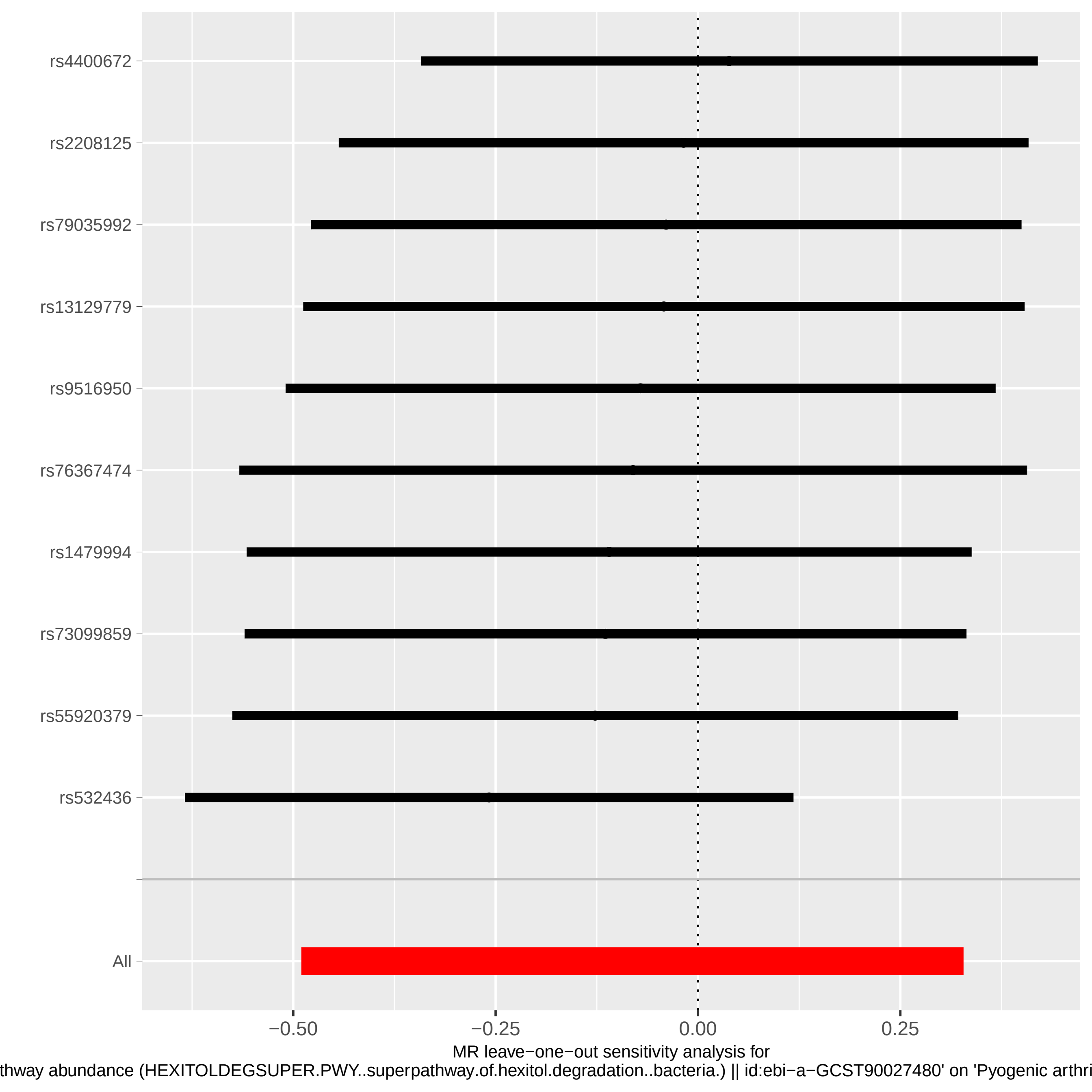
rs4400672
rs2208125
rs79035992
rs13129779
rs9516950
rs76367474
rs1479994
rs73099859
rs55920379
rs532436
All
−0.50
−0.25
0.00
0.25
MR leave−one−out sensitivity analysis for
thway abundance (HEXITOLDEGSUPER.PWY..superpathway.of.hexitol.degradation..bacteria.) || id:ebi−a−GCST90027480' on 'Pyogenic arthri
